# The Hypocholesterolemic Effects of* Eryngium carlinae* F. Delaroche Are Mediated by the Involvement of the Intestinal Transporters ABCG5 and ABCG8

**DOI:** 10.1155/2017/3176232

**Published:** 2017-12-14

**Authors:** Ibrahim Guillermo Castro-Torres, Minarda De la O-Arciniega, Elia Brosla Naranjo-Rodríguez, Víctor Alberto Castro-Torres, Miguel Ángel Domínguez-Ortíz, Mariano Martínez-Vázquez

**Affiliations:** ^1^Instituto de Química, Universidad Nacional Autónoma de México (UNAM), Ciudad de México, Mexico; ^2^Área Académica de Farmacia, Instituto de Ciencias de la Salud, Universidad Autónoma del Estado de Hidalgo, Pachuca de Soto, HGO, Mexico; ^3^Departamento de Farmacia, Facultad de Química, UNAM, Ciudad de México, Mexico; ^4^Laboratorio de Productos Naturales, Instituto de Ciencias Básicas, Universidad Veracruzana, Xalapa de Enríquez, VER, Mexico

## Abstract

Hypercholesterolemia is a metabolic disorder characterized by a high concentration of cholesterol in the blood.* Eryngium carlinae* is a medicinal plant used to treat lipid diseases. The goal of this work was to evaluate, in a model of hypercholesterolemia in mice, the hypocholesterolemic effect of a hydroalcoholic extract of* E. carlinae* and its main metabolite, D-mannitol. Biochemical analyses of serum lipids and hepatic enzymes were performed by photocolorimetry. We performed histopathological studies of the liver and the expression of the intestinal cholesterol transporters Abcg5 and Abcg8 was determined by standard western blot method. Our results showed that hydroalcoholic extract at doses of 100 mg/kg and D-mannitol at doses of 10 mg/kg reduced the concentration of both total cholesterol and non-HDL cholesterol, without altering the concentration of HDL cholesterol and without damage to hepatocytes. Treatment with the extract increased Abcg8 intestinal transporter expression, while D-mannitol decreased the expression of the two Abcg5/Abcg8 transporters, compared with the hypercholesterolemic group. Considering that Abcg5/Abcg8 transporters perform cholesterol efflux, our results demonstrate that the lipid-lowering effect of the hydroalcoholic extract may be associated with the increase of Abcg8 expression, but the hypocholesterolemic effect of D-mannitol is independent of overexpression of these intestinal transporters and probably they have another mechanism of action.

## 1. Introduction

Hypercholesterolemia is a metabolic disorder characterized by an increase in the concentration of plasma cholesterol (above 200 mg/dL); it is considered the primary risk factor for developing cardiovascular disease (atherosclerosis) [1, 2]. Hypercholesterolemia can be classified as primary if this is associated with congenital problems or improper food habits. When the hypercholesterolemia is associated with some disease such as diabetes mellitus, acute renal failure, or liver failure or the intake of types of drugs it is classified as secondary. Primary hypercholesterolemia is the most prevalent [3]. HMG-CoA reductase inhibitors (“statins”) represent the most effective and widely prescribed drugs currently available for the reduction of low-density lipoprotein cholesterol. Although in general these drugs have proven their therapeutic value adverse events have been reported. Skeletal muscle-related events are the most common adverse events of statin treatment [4]. There has also been an increase in the number of reports of suspected psychiatric adverse reactions associated with statins [5]. Furthermore, statins may partially operate by lowering testosterone [5].

Ezetimibe is another popular hypolipidemic agent. This drug inhibits the intestinal absorption of cholesterol from dietary and biliary sources by impeding the transport of cholesterol across the intestinal wall [6]. Ezetimibe does not affect the absorption of bile acids, fatty acids, fat-soluble vitamins, or triglycerides. However, it inhibits the expression of the Niemann-Pick C1L1 protein [7]. Nevertheless, there are reports concerning alleged psychiatric unfavorable reactions associated with ezetimibe [5].

Due to side effects of these drugs other therapeutic agents have been sought. Among them are the so-called medicinal plants. For example, the Malaysian medicinal plant* Hibiscus rosa-sinensis *reduces blood pressure and cholesterol concentration in blood. This is due to the existence of saponins in this species [8]. Saponins bind to cholesterol to form insoluble complexes excreted via the bile. This prevents cholesterol reabsorption and results in a reduction of serum cholesterol [9].* Camellia japonica* and* Amaranthus viridis* have also demonstrated important lipid-lowering effects in experimental models [10, 11].

In the Mexican traditional medicine, there are two species,* Eryngium heterophyllum* and* E. carlinae* Delaroche which both under the trivial name of “toad herb” have a great ethnopharmacological tradition to relieve some gastrointestinal diseases and to treat hypercholesterolemia [12]. An exhaustive search of the literature indicates a single report linking* E. carlinae* with hypolipidemic activity. This report demonstrates that an ethanolic extract of this species reduced the levels of creatinine, uric acid, total cholesterol, and triglycerides, parameters that are exacerbated in diabetes, in streptozotocin-induced diabetic rats. However, no toxicity data or phytochemical analyses were reported. Neither of the rats fed hypercholesterolemic diets was treated with the extract [13]. On the other hand, it is known that Abcg5 and Abcg8 proteins are responsible for mediating cholesterol efflux [14]. Therefore, inhibition of these proteins could trigger a hypercholesterolemia process [14].

Considering the above, we decide to evaluate the effectiveness of a hydroalcoholic extract of* Eryngium carlinae* (ECHE) and its major metabolite, D-mannitol, as hypocholesterolemic agents in hypercholesterolemic mice. We also studied the possible involvement of the intestinal proteins Abcg5 and Abcg8 in the mechanism of action of these drugs.

## 2. Material and Methods

### 2.1. Animal Experimental Protocol

Male CD1 mice (30 ± 2 g, 8–10 weeks old) were used (Harlan Laboratories, Mexico). Animals were housed in plastic cages in a temperature-controlled room with 12 : 12 h light-dark cycles and provided ad libitum with access to food and water. After one week of acclimatization, the animals were randomly divided into 7 experimental treatments groups (*n* = 6) (Table 1). For eight weeks, group 1 (control group) was maintained on a regular chow diet (Laboratory Rodent Chow 5001, Harlan Laboratories, Mexico).

The other six groups were feeding with a diet enriched with 1% cholesterol and 0.5% cholic acid (hypercholesterolemic/lithogenic diet, Harlan Laboratories TD 03451, 1% cholesterol, 0.5% sodium cholate). The mice had free access to this diet for four weeks. After this period, we found that the mice were already hypercholesterolemic, compared with healthy mice. At this point, the different treatments were administered orally, every day at 15:00 h, for the rest of the four weeks along with the hypercholesterolemic diet. Animal care and procedures were conducted according to the guidelines approved by Norma Oficial Mexicana (NOM-062-ZOO-1999) and were subjected to experimental protocols approved by the Comité Interno Ético para el Uso y Cuidado de los Animales de Laboratorio (15-CIECUAL, February 2017), Instituto de Ciencias de la Salud, Universidad Autónoma del Estado de Hidalgo.

### 2.2. Chronic Toxicity Test

The chronic toxicity test was performed using female CD1 mice (30 ± 2 g in weight and approximate age of 8–10 weeks), which were given orally the alcoholic extract (ECHE), D-mannitol, or hexa-O-acetyl-D-mannitol under the same schedule as the hypercholesterolemia study.

### 2.3. Biochemical Analysis

After eight weeks, the animals of all groups were fasted, weighed, and anesthetized (sodium pentobarbital 70 mg/kg). The blood samples were drawn from the retroorbital vein; then the mice were sacrificed under anesthesia. Serum was obtained from blood samples by centrifugation, and the different biochemical parameters were measured. Biochemical analysis was carried into a Randox Daytona automate using a commercial kit (total cholesterol CH201, 6 × 100 mL; HDL cholesterol-direct clearance method, CH1383,* R*1 3 × 2.5 L* R*2 1 × 2.5 L; LDL cholesterol CH2656* R*1 6 × 78 mL* R*2 3 × 52 mL and glucose, GOD-PAP, and hexokinase, GL 364 10 × 100 mL) according to the manufacturer's protocol.

We reported total cholesterol, HDL, and non-HDL cholesterol concentrations (Enzymatic Methods, Accelab, Mexico). In chronic toxicity test, we quantified the concentration of aspartate transaminase (AST) and alanine transaminase (ALT) liver enzymes, as well as the total cholesterol concentration (Accelab, Mexico).

### 2.4. Histopathological Study

Livers were entirely randomly selected and fixed in 4% neutral buffered formaldehyde embedded in paraffin, and 5 *μ*m thin slices were cut and stained with hematoxylin-eosin. A pathologist performed the histological studies.

### 2.5. Molecular Analysis

The first 5 centimeters of the small intestine corresponding to the jejunum were used. We analyze this inner region because it is the site where cholesterol is absorbed. The intestinal tissue was homogenized by mixing it with a protease inhibitor, and the cells were subsequently lysed and centrifuged. The quantified protein concentration was performed by the Lowry method.

The expression of major intestinal cholesterol transporters in mice, Abcg5 and Abcg8, was analyzed by the standard western blot method. We perform 4 replicates of these molecular analyses. Antibodies were purchased from Santa Cruz Biotechnology, Inc., México: ABCG5 (H-300): sc-25796, rabbit polyclonal; ABCG8 (H-300): sc-30111, rabbit polyclonal and b-actin (C4); HRP: sc-47778, mouse monoclonal. We used 12% SDS-polyacrylamide gel and proteins were transferred to PVDF membranes. Membranes were incubated in blocking buffer. Primary antibodies were diluted as 1 : 100 in blocking buffer and incubated overnight at 4°C. Secondary antibody was diluted as 1 : 2000 and coupled with horseradish peroxidase. Proteins were visualized by chemiluminescence and our results were normalized with *β*-actin.

### 2.6. General Experimental Procedures

NMR spectra were acquired in CDCl_3_ at room temperature on a Bruker-Fourier (A300 MHz) spectrometer and the chemical shifts are given as *δ* values with reference to tetramethylsilane (TMS) as the internal standard.

### 2.7. Plant Material

Aerial parts of* E. carlinae* Delaroche (Apiaceae) were collected near Atlixco, Puebla, Mexico, in July 2011. The plant was dried, ground, and stored in the refrigerator at 0°C until it was used. The species was taxonomically identified by Dr. Isolda Luna and a voucher (097586) has been deposited at Herbario de la Facultad de Ciencias, México (FCME).

### 2.8. Extraction and Isolation

The air-dried and ground aerial parts of* E. carlinae* (1000 g) were extracted with a mixture of EtOH-H_2_0 (7 : 3) (4 L) at 60°C under constant shaking for 2 h. The solvent was filtered and evaporated under reduced pressure to give 10.55 g of hydroalcoholic extract (ECHE).

### 2.9. D-Mannitol

Treatment of ECHE (4 g) with MeOH (50 mL) afforded a solid and a solution. The solution was filtered and mixed with activated carbon and subsequently gently heated. The solution was filtered using a Büchner funnel with celite pad, and it was evaporated under reduced pressure to give a solid which was purified by crystallization method with MeOH to give 650 mg of D-mannitol. The identity of this metabolite was achieved by comparison of its spectroscopic and spectrometric data with those reported in the literature [15].

### 2.10. Hexa-O-acetyl-D-mannitol

The residue insoluble in MeOH (510 mg) was dissolved in dry pyridine (3 mL) and Ac_2_O (3 mL) was added. The reaction mixture was heated at 60°C during 4 h. Usual work-up provided a residue which was chromatographed on a silica gel open column using* n*-hexane-EtOAc solvent mixture of increasing polarity. Hexa-O-acetyl-D-mannitol was isolated from the fractions eluted with 9 : 1, hexane : EtOAc. The identity of the acetate derivate was achieved by comparison of its spectroscopic and spectrometric data with those reported in the literature [16].

### 2.11. Statistical Analysis

The statistical analyses were performed using a Sigma Plot program. Data were analyzed using one-way analysis of variance (one-way ANOVA) and Tukey's post hoc test. Each point in the tables and figures represents the mean ± standard error.

## 3. Results

### 3.1. Phytochemistry

Treatment of ECHE with methanol yielded both a methanol solution and a methanol-insoluble solid; the latter phase was the most abundant. D-Mannitol was isolated from the methanol soluble fraction by evaporation of the solvent under reduced pressure and treatment of the residue with methanol. The methanol-insoluble solid (510 mg) was dissolved in pyridine and treated with acetic anhydride to yield 670 mg of acetylation product. The chromatographic separation of this product yielded 135 mg of hexa-O-acetyl-D-mannitol. The identification of the D-mannitol and its acetylated derivative was achieved by comparison of its spectral and physical data with those in literature [15, 16].

### 3.2. Chronic Toxicity

The mice showed no evident signs of toxicity (motor incoordination, piloerection, dilation of pupils, etc.) after being given both 100 and 500 mg/kg doses of the extract for 4 weeks. However, the hydroalcoholic extract caused liver toxicity in mice at doses of 500 mg/kg, as evidenced by an increase in the concentration of aspartate transaminase (AST) and alanine transaminase (ALT) compared to the control group (Table 2). On the other hand, D-mannitol and its derivative caused a slight increase in the concentration of liver enzymes; however, these effects were less toxic than that exerted by the extract at 500 mg/kg (Table 2). Additionally, the extract and the D-mannitol as well as its acetyl derivative did not alter the concentration of cholesterol; the values obtained in the different treatments can be compared with values obtained with normocholesterolemic mice (Table 2).

### 3.3. Cholesterol Concentration

For eight weeks mice had free access to cholesterol-rich diet. However, by the fourth week, according to our analysis, the animals were already hypercholesterolemic. Considering the above, we decided to start the administration of the different treatments along with the cholesterol-rich diet to the hypercholesterolemic mice at the fourth week and finish it until the eighth week. The mice that received the high cholesterol diet showed an increase in both total cholesterol levels and the non-HDL fraction, as well as a significant decrease in HDL cholesterol compared with control mice (Table 3). The mice treated with ezetimibe (10 mg/kg), ECHE (100 and 500 mg/kg), and D-mannitol (10 mg/kg) significantly showed a reduction in the concentration of both total cholesterol and non-HDL cholesterol compared to the group that developed hypercholesterolemia. The latter revealed a total cholesterol concentration over 200 mg/dL. On the other hand, treatments with ezetimibe, D-mannitol, and ECHE at 100 mg/kg doses caused an increase in HDL cholesterol levels compared to the hypercholesterolemic group. Although the hexa-O-acetyl-D-mannitol derivate also decreased the total cholesterol this reduction is not as pronounced as the nonacetylated compound. According to our results, the acetylated derivative preferably decreases the non-HDL concentration not that of HDL (Table 3).

### 3.4. Histopathology

**Figure 1:**
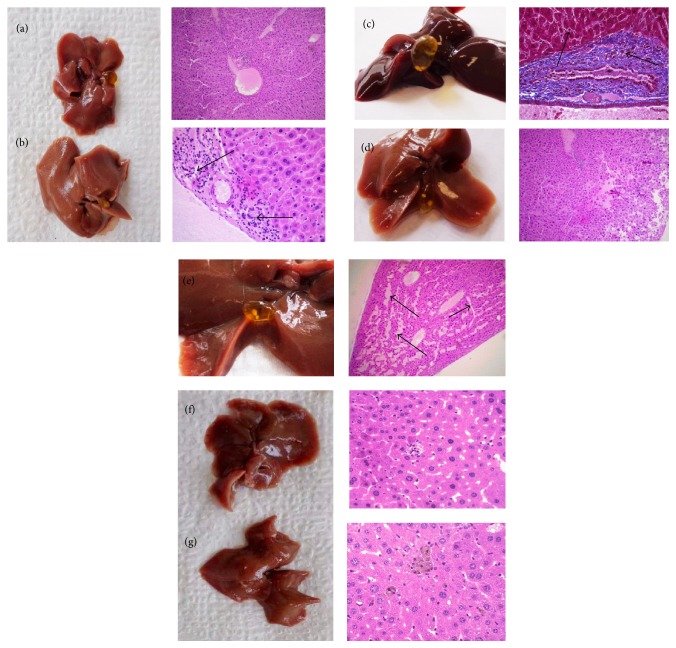
Histopathological microscopic examination in mice liver tissues. Control (a). Hypercholesterolemia (b). Ezetimibe 10 mg/kg (c). ECHE, 100 mg/kg (d). ECHE, 500 mg/kg (e). Hexa-O-acetyl-D-mannitol 10 mg/kg (f). D-Mannitol, 10 mg/kg (g). There were no apparent histological changes in control group fed with a regular chow diet (Figure 1(a)). In Figure 1(b), black arrows indicate the epithelial hyperplasia and granulocyte infiltration in liver of CD1 mice after being fed with a hypercholesterolemic diet. In Figure 1(c) the liver treated with ezetimibe also presented inflammatory infiltration and epithelial hyperplasia; black arrows indicate hepatocellular necrosis. In Figure 1(d) there were no apparent changes after treatment with ECHE 100 mg/kg. In Figure 1(e) all liver tissues showed fibrosis bands (black arrows) and epithelial hyperplasia. There were no apparent histological changes in groups treated with hexa-O-acetyl-D-mannitol (Figure 1(f)) or D-mannitol (Figure 1(g)).

Histopathology results were compared between different experimental groups (Figure 1). Our results showed that the liver tissues of normal diet mice group presented a moderate congestion in hepatocytes (10x magnification) (Figure 1(a)). Conversely, the liver tissues of the hypercholesterolemic group showed diffuse moderate hepatocellular degeneration, periportal inflammation in blood vessels, and damage to endothelium of sinusoids caused by a homogenous acellular material like fibrin (40x magnification) (Figure 1(b)). The liver samples of the ezetimibe group showed moderate chronic diffuse liver congestion with periportal fibrosis and there was moderate intrahepatic cholestasis (40x magnification) (Figure 1(c)). Almost the same results as the normal group were observed with the group treated at 100 mg/kg doses of extract (10x magnification) (Figure 1(d)). Although the treatment with extract at 500 mg/kg doses induced serum cholesterol levels, it produced damage in the liver. The liver samples of the latter group showed hepatocellular necrosis, moderate intrahepatic cholestasis, and hepatocellular degeneration (40x magnification) (Figure 1(e)). There were no apparent histological changes in groups treated with hexa-O-acetyl-D-mannitol (40x magnification) (Figure 1(f)) or D-mannitol (40x magnification) (Figure 1(g)).

### 3.5. Molecular Analysis

**Figure 2:**
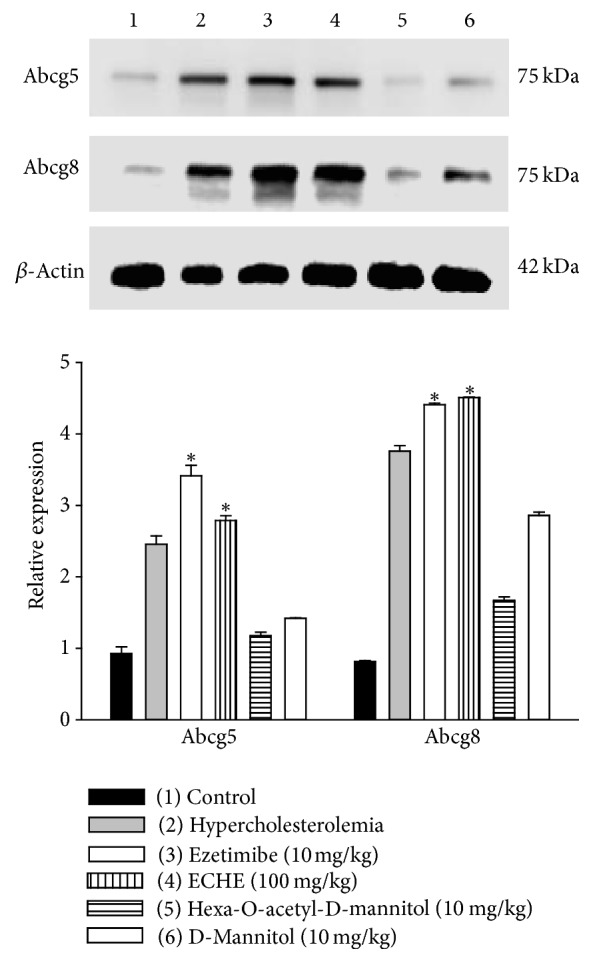
Western blot results. Western blot analysis of Abcg5 and Abcg88 in small intestine tissues of mice. Control (1), hypercholesterolemia (2), ezetimibe 10 mg/kg (3), ECHE 100 mg/kg (4), hexa-O-acetyl-D-mannitol (5), and D-mannitol (6). Total protein lysates were separated by 12–15% SDS-PAGE followed by western blot analysis using the indicated antibodies. *β*-Actin was used as a loading control for total cell extracts. Densitometry of western blots for ABCG5 and ABCG8, ^*∗*^*p* < 0.05 comparing groups treated with ezetimibe (10 mg/kg) and ECHE (100 mg/kg) with hypercholesterolemic group.

Since ECHE (100 mg/kg) showed a similar hypocholesterolemic activity compared to ECHE (500 mg/kg) without the induction of hepatotoxicity, only ECHE (100 mg/kg) was analyzed by western blot technique. Expression of Abcg8 intestinal transporter increased after treatments with hydroalcoholic extract at 100 mg/kg dose and with ezetimibe at doses of 10 mg/kg (Figure 2). These two treatments (ezetimibe and extract) appeared to increase the expression of Abcg5 and Abcg8 in the small intestine. On the other hand, the administration of D-mannitol and its hexa-acetyl derivative decreased the expression of Abcg5/Abcg8 proteins, compared with hypercholesterolemic group.

## 4. Discussion

It has been proposed that two ATP-binding cassette (ABC) transporters, Abcg5 and Abcg8, restricted sterol absorption and promoted biliary sterol excretion in humans. Furthermore, it has been reported that increased expression of* ABCG5* and* ABCG8* genes selectively drives biliary neutral sterol secretion and reduces intestinal cholesterol absorption [17, 18].

Taking the above into account, we studied the possible participation of these proteins in the antihyperlipidemic activity of ECHE, D-mannitol, and its hexa-acetate derivative in hypercholesterolemic mice.

The effect of dietary cholesterol on the expression of Abcg5 and Abcg8 in the small intestine is controversial [19]. Some studies in mice have shown that dietary supplementation of cholesterol increases the expression of Abcg5 and Abcg8 in the small intestine while other authors claimed that fat diet did not show any effect on the expression of these transporters [17, 20]. Nevertheless, in our case, an overexpression of ABCG5/ABCG8 proteins was observed in the hypercholesterolemic group compared with the control group (Figure 2). Our results showed that ECHE at 100 mg/kg doses and ezetimibe at 10 mg/kg doses caused an increase in the expressions of the ABCG8 and ABCG5 transporters (Figure 2). It has been reported that the ABCG5/ABCG8 expression in both the liver and intestine protects animals from sterol accumulation, and intestinal ABCG5/ABCG8 contributes to extrahepatic cholesterol efflux in mice [18]. Jia and coworkers demonstrated, in knockout mice, that ABCG5/ABCG8 deficiency reduces the uptake and secretion of both dietary triglycerides and cholesterol by the intestine [21]. Therefore, our results suggested that ABCG8 could be involved in the hypolipidemic activities of ECHE.

However, D-mannitol and the hexa-O-acetyl-D-mannitol not only hindered overexpression of ABCG5/ABCG8 but also inhibited the overexpression of these proteins induced by the hypercholesterolemic diet (Figure 2). These results suggest that the antihypercholesterolemic properties of D-mannitol seem to be independent of ABCG5/ABCG8 overexpression.

Under physiological conditions, dietary cholesterol is efficiently solubilized by bile salt-phospholipid mixtures in the form of either mixed micelles or vesicles that formed in bile [22]. Bile flow is generated by transepithelial transport of water and ionic/nonionic solutes via transcellular and paracellular pathways mainly driven by osmotic pressure [23]. D-Mannitol is the most widely used osmotic diuretic and can be absorbed solely by the paracellular route [24]; therefore, in our experimental study, this molecule could favor the osmotic pressure for proper regulation of bile composition. By having mixed micelles in the bile, it is possible to favor the adequate transport of cholesterol, preventing it from accumulating in the enterocytes and avoiding the development of hypercholesterolemia.

The latter result is a little surprising if we consider that the ECHE induced overexpression of the enzymes ABCG5/ABCG8. However, there are several reports about the isolates from plants that lowered plasma cholesterol by increasing fecal excretion of acidic and neutral sterols in mice fed a cholesterol-enriched diet accompanied with downregulation of gene expression of intestinal ABCG8 [23–26].

## 5. Conclusion

In conclusion, we showed that ECHE (100 mg/kg) and D-mannitol (10 mg/kg) exert, each, hypolipidemic effects in hypercholesterolemic mice, with similar activity compared to ezetimibe (10 mg/kg), without causing damage to liver and without affecting the levels of HDL cholesterol. The hypocholesterolemic effect of ECHE seems to be mediated by ABCG5/ABCG8 intestinal transporters.

## Figures and Tables

**Table 1:** Experimental groups.

Number	Experimental group	Treatment
(1)	Control	Standard diet (Laboratory Rodent Diet 5001) ad libitum
(2)	Hypercholesterolemia	Hypercholesterolemic diet (1% cholesterol, 0.5% cholic acid) ad libitum
(3)	Ezetimibe	Ezetimibe 10 mg/kg and hypercholesterolemic diet ad libitum (Merck/Schering-Plough Pharmaceuticals, USA)
(4)	Hydroalcoholic extract	Extract of *Eryngium carlinae* at a dose of 100 mg/kg and hypercholesterolemic diet and water ad libitum
(5)	Hydroalcoholic extract	Extract of *Eryngium carlinae* at a dose of 500 mg/kg and hypercholesterolemic diet ad libitum
(6)	Hexa-O-acetyl-D-mannitol	Mannitol hexaacetate at a dose of 10 mg/kg and hypercholesterolemic diet ad libitum
(7)	D-Mannitol	D-Mannitol at a dose of 10 mg/kg and hypercholesterolemic diet ad libitum

Different treatments during the evaluation of *Eryngium carlinae*. All treatments were administered every day at 15:00 h for four weeks.

**Table 2:** Analysis of biochemical parameters in toxicity test.

Number	Experimental group	Total cholesterol (mg/dL)	ALT (IU/L)	AST (IU/L)
(1)	Control	125.1 ± 2.1	84.3 ± 1.3	42.2 ± 0.5
(2)	ECHE (100 mg/kg)	129.6 ± 1.2	92.6 ± 0.6	46.3 ± 0.8
(3)	ECHE (500 mg/kg)	120.2 ± 3.3	159.5 ± 4.8^*∗*^	182.2 ± 2.6^*∗*^
(4)	Hexa-O-acetyl-D-mannitol (10 mg/kg)	130.1 ± 3.3	84.7 ± 0.5	53.3 ± 0.8
(5)	D-Mannitol (10 mg/kg)	127.1 ± 3.7	91.9 ± 0.8	58.5 ± 0.4

^*∗*^Values indicate significant statistical difference (*p* < 0.05) between groups treated with extracts and metabolites versus control group. One-way ANOVA with Tukey's post hoc test. Values are reported as mean ± standard error using six mice in each experimental group.

**Table 3:** Analysis of serum cholesterol.

Number	Experimental group	Cholesterol concentration (mg/dL)		
		Total	HDL	Non-HDL
(1)	Control	148.3 ± 4.4	126.5 ± 4.8	21.8 ± 0.7^*∗*^
(2)	Hypercholesterolemia	223.5 ± 2.6	97.5 ± 5.6	132.8 ± 4.8
(3)	Ezetimibe (10 mg/kg)	128.1 ± 1.8^*∗*^	119.8 ± 2.8^*∗*^	10.5 ± 0.7^*∗*^
(4)	ECHE (100 mg/kg)	139.7 ± 2.8^*∗*^	120.9 ± 3.7^*∗*^	17.1 ± 0.6^*∗*^
(5)	ECHE (500 mg/kg)	129.6 ± 2.9^*∗*^	112.5 ± 3.1	17.2 ± 0.9^*∗*^
(6)	Hexa-O-acetyl-D-mannitol (10 mg/kg)	198.1 ± 4.9	98.1 ± 3.2	93.7 ± 2.2^*∗*^
(7)	D-Mannitol (10 mg/kg)	137.6 ± 3.3^*∗*^	117.9 ± 2.7^*∗*^	19.1 ± 1.5^*∗*^

^*∗*^Values indicate significant statistical difference (*p* < 0.05) between groups with different treatments versus group treated with hypercholesterolemic diet. One-way ANOVA with Tukey's post hoc test. Values are reported as mean ± standard error using six mice in each experimental group.
